# Clinical Experience with Ceftazidime-Avibactam for the Treatment of Infections due to Multidrug-Resistant Gram-Negative Bacteria Other than Carbapenem-Resistant *Enterobacterales*

**DOI:** 10.3390/antibiotics9020071

**Published:** 2020-02-09

**Authors:** Antonio Vena, Daniele Roberto Giacobbe, Nadia Castaldo, Annamaria Cattelan, Cristina Mussini, Roberto Luzzati, Francesco Giuseppe De Rosa, Filippo Del Puente, Claudio Maria Mastroianni, Antonio Cascio, Sergio Carbonara, Alessandro Capone, Silvia Boni, Chiara Sepulcri, Marianna Meschiari, Francesca Raumer, Alessandra Oliva, Silvia Corcione, Matteo Bassetti, for the Ceftabuse Study Group

**Affiliations:** 1 Department of Health Sciences, Infectious Disease Clinic, University of Genoa, Genoa 16132, Italy; anton.vena@gmail.com (A.V.); daniele.roberto.giacobbe@edu.unige.it (D.R.G.); fildelp@gmail.com (F.D.P.); chiara.sepulcri@gmail.com (C.S.); 2 Hospital Policlinico San Martino−IRCCS, Genoa 16132, Italy; 3 Infectious Diseases Clinic, Department of Medicine University of Udine and Azienda Sanitaria Universitaria Integrata, Udine 33100, Italy; nadiacastaldo.nc@gmail.com; 4 Infectious Diseases Unit, Department of Internal Medicine, Azienda Ospedaliera−Universitaria di Padova, Padua 35128, Italy; annamaria.cattelan@aopd.veneto.it (A.C.); francesca.raumer@gmail.com (F.R.); 5 Infectious Diseases Clinics, University of Modena and Reggio Emilia, Modena 41121, Italy; crimuss@unimore.it (C.M.); mariannameschiari1209@gmail.com (M.M.); 6 Infectious Diseases Unit, University Hospital of Trieste, Trieste 34127, Italy; roberto.luzzati@asuits.sanita.fvg.it; 7 Department of Medical Sciences, Infectious Diseases, University of Turin, Turin 10124, Italy; francescogiuseppe.derosa@unito.it (F.G.D.R.); corcione.silvia@gmail.com (S.C.); 8 Department of Anesthesiology, Critical Care Medicine and Pain Therapy, Sapienza University of Rome, Rome 00185, Italy; claudio.mastroianni@uniroma1.it (C.M.M.); alessandra.oliva@uniroma1.it (A.O.); 9 Department of Health Promotion Sciences, Mother and Infant Care, Internal Medicine and Medical Specialties, University of Palermo, Palermo 90133, Italy; antonio.cascio03@unipa.it; 10 Clinic of Infectious Diseases, University Hospital of Bari Consorziale Policlinico, Bari 70121, Italy; s_carbonara@yahoo.it; 11 Clinical Department, National Institute for Infectious Diseases “L. Spallanzani”, Rome 00149, Italy; alessandrocapone@ymail.com; 12 Divisione di Malattie Infettive, Ospedale Sant’Andrea, La Spezia 19121, Italy; silvia.boni@asl5.liguria.it

**Keywords:** carbapenem-sparing regimen, ceftazidime-avibactam, Pseudomonas aeruginosa, ESBL-producing Enterobacterales, nosocomial pneumonia

## Abstract

Background: Experience in real clinical practice with ceftazidime-avibactam for the treatment of serious infections due to gram−negative bacteria (GNB) other than carbapenem-resistant *Enterobacterales* (CRE) is very limited. Methods: We carried out a retrospective multicenter study of patients hospitalized in 13 Italian hospitals who received ≥72 h of ceftazidime-avibactam for GNB other than CRE to assess the rates of clinical success, resistance development, and occurrence of adverse events. Results: Ceftazidime-avibactam was used to treat 41 patients with GNB infections other than CRE. Median age was 62 years and 68% of them were male. The main causative agents were *P. aeruginosa* (33/41; 80.5%) and extended spectrum beta lactamase (ESBL)-producing *Enterobacterales* (4/41, 9.8%). Four patients had polymicrobial infections. All strains were susceptible to ceftazidime-avibactam. The most common primary infection was nosocomial pneumonia (*n* = 20; 48.8%), primary bacteremia (*n* = 7; 17.1%), intra-abdominal infection (*n* = 4; 9.8%), and bone infection (*n* = 4; 9.8%). Ceftazidime-avibactam was mainly administered as a combination treatment (*n* = 33; 80.5%) and the median length of therapy was 13 days. Clinical success at the end of the follow-up period was 90.5%, and the only risk factor for treatment failure at multivariate analysis was receiving continuous renal replacement therapy during ceftazidime-avibactam. There was no association between clinical failures and type of primary infection, microbiological isolates, and monotherapy with ceftazidime-avibactam. Only one patient experienced recurrent infection 5 days after the end of treatment. Development of resistance to ceftazidime-avibactam was not detected in any case during the whole follow-up period. No adverse events related to ceftazidime-avibactam were observed in the study population. Conclusions: Ceftazidime-avibactam may be a valuable therapeutic option for serious infections due to GNB other than CRE.

## 1. Introduction

The increasing incidence of infections caused by multidrug-resistant gram-negative bacteria (MDR-GNB), such as *Pseudomonas aeruginosa*, *Acinetobacter baumannii,* or members of the order *Enterobacterales*, has dramatically hindered the selection of an appropriate antimicrobial therapy, leading to an increase in morbidity and mortality in patients with such infections [1,2,3,4].

Ceftazidime-avibactam is a new β-lactam/β-lactamase inhibitor currently approved by the European Medicines Agency for the treatment of complicated intra-abdominal infections [5,6], complicated urinary tract infections [7], hospital-acquired pneumonia (including ventilator-associated pneumonia), and more generally, for aerobic gram-negative infections with limited treatment options [8]. In real-life experiences, high rates of favorable response to ceftazidime-avibactam treatment are reported in patients with infections due to carbapenem−resistant *Enterobacterales* (CRE), with an overall success rate of about 70% [9,10,11,12,13,14,15], whereas post-marketing experience regarding the use of ceftazidime-avibactam for infections due to MDR-GNB other than CRE remains scarce [16,17,18] Moreover, information regarding features associated with clinical failures and the emergence of resistance in this group of patients are even scarcer. For this reason, in this multicenter study we describe our experience about the use of ceftazidime-avibactam for the treatment of infections due to MDR-GNB other than CRE in 13 Italian hospitals. More specifically, the primary objective of the study was to describe the rate of clinical cure in the study population. The secondary objectives were to describe: (i) the characteristics of patients who experienced clinical failure; (ii) resistance development rate; (iii) adverse events (AE) related to ceftazidime-avibactam treatment.

## 2. Results

### 2.1. Baseline Characteristics

A total of 41 consecutive patients treated with ≥72 h of ceftazidime-avibactam for MDR-GNB infections other than CRE were included in the study. Their baseline characteristics are presented in Table 1. Their median age was 62 years (interquartile range (IQR) 41–70) and 68% (28/41) were male. The most common underlying condition was cardiovascular disease (*n* = 14, 34.1%) followed by chronic renal failure (*n* = 9, 22.0%). In 34 patients (82.9%) more than one underlying disease was present, and the median Charlson comorbidity index was 4 (IQR 2–6). As many as 24 patients (58.5%) presented with sepsis or septic shock and 10 of them were admitted to the intensive care unit (ICU) due to the gram-negative infection.

Types of primary infection and causative microorganisms are presented in Table 2. Overall, nosocomial pneumonia (*n* = 20; 48.8%), primary bacteremia (*n* = 7; 17.1%), intra-abdominal infection (*n* = 4; 9.8%), and bone infection (*n* = 4; 9.8%) were the most common types of infection. Overall 65% (13/20) and 35% (7/20) of nosocomial pneumonia cases were ventilator associated and hospital acquired, respectively. 

### 2.2. Microbiology

Thirty-seven of the 41 episodes were monomicrobial, whereas four were caused by more than one MDR-GNB, leading to a total of 45 isolates from 41 patients. As shown in Table 3, isolated organisms were *P. aeruginosa* (*n* = 38) and *Enterobacterales* (*n* = 7). All *Enterobacterales* isolates were phenotypically classified as extended spectrum beta lactamase (ESBL)-producing strains.

Almost 90% of isolates were non-susceptible to cefepime, ceftazidime, ciprofloxacin, and piperacillin-tazobactam. In addition, 11% of isolates were non-susceptible to ceftolozane-tazobactam and 26.6% were non-susceptible to colistin. According to their susceptibility profiles, 11 isolates (24.4%) were classified as MDR, 25 (55.6%) as extremely drug resistant (XDR), and 9 (20.0%) as pandrug resistant (PDR).

### 2.3. Characteristics of Ceftazidime-Avibactam Therapy

Twenty−seven patients (65.9%) received ceftazidime-avibactam as secondary therapy with the median time for switching to ceftazidime-avibactam as 11 days of treatment with other agents (IQR 4.5–13 days) (Table 4). Piperacillin-tazobactam (48.1%, (13/27)), carbapenems (25.9%, (7/27)), and colistin (22.2%; (6/27)) were the three most common antimicrobials prescribed prior to initiation of ceftazidime-avibactam.

The main reason for switching to ceftazidime-avibactam was antimicrobial resistance to previous antibiotic therapy in 25/41 patients (61.0%) and failure of previous antibiotic treatment in 14/41 patients (34.1%). Most patients received ceftazidime-avibactam treatment in combination with other antibiotics (*n* = 33; 80.5%), that mainly included intravenous colistin (*n* = 12), aminoglycosides (*n* = 11), carbapenems (*n* = 5), or fosfomycin (*n* = 5). The median duration of ceftazidime-avibactam treatment was 13 (range 3–49) days.

Source control of infection was necessary in 13/41 patients (31.7%) and adequate in 11 of them (84.6%).

### 2.4. Clinical Cure

**Figure 1:**
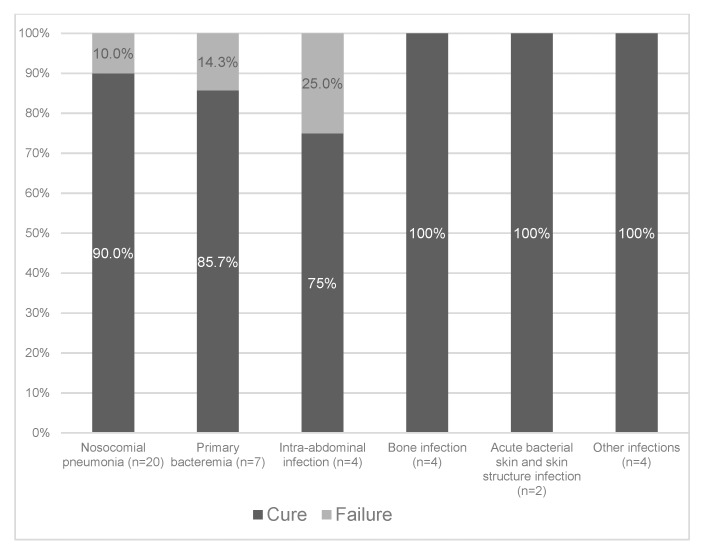
Clinical cure rates according to different types of infection.

**Figure 2:**
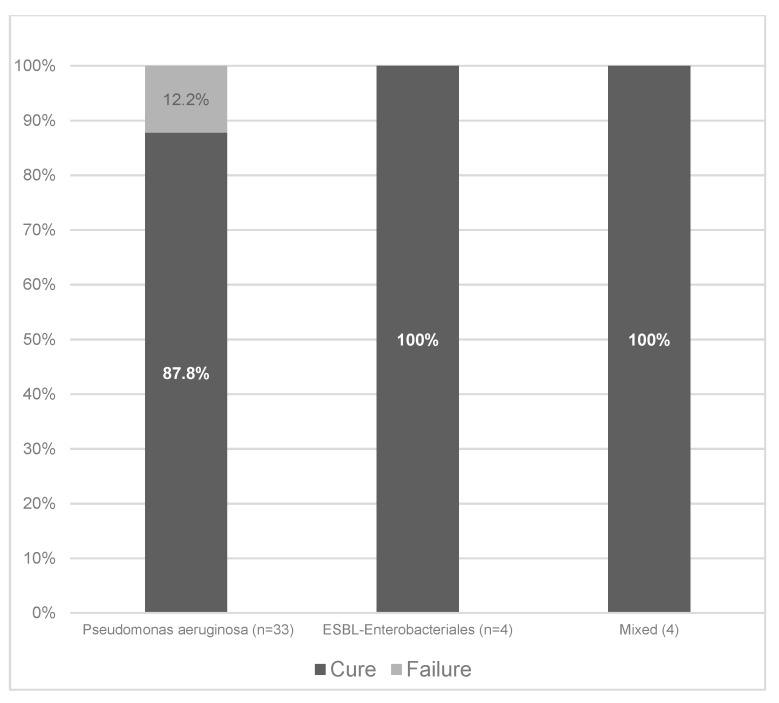
Clinical cure rates according to the different causative agents.

Among the 41 patients treated with ceftazidime-avibactam, clinical cure was achieved in 37 (90.2%). Clinical cure rates stratified according to the different types of infection are shown in Figure 1. With regard to the two most common types of infection, clinical cure was achieved in 90% of patients with nosocomial pneumonia (18/20) and 86% of patients with primary bacteremia (6/7). Stratification of clinical cure rates according to the causative microorganisms is presented in Figure 2. The clinical cure rates were 87.8%, 100%, and 100%, in patients with *P. aeruginosa* (29/33), ESBL-producing *Enterobacterales* (4/4), and polymicrobial infection (4/4), respectively.

### 2.5. Risk factors for Clinical Failures

Details of the four patients who experienced clinical failure are presented in Table 5 and Supplementary Materials.

In order to identify predictors of treatment failure, univariate and multivariate analyses were performed after adjusting for confounding factors (Table 6). The only factor related to clinical failure was receipt of continuous renal replacement therapy at the time of infection onset (odds ratio (OR) 29.03, 95% CI 1.69–498.35; *p* = 0.02).

### 2.6. Adverse Events

Development of resistance to ceftazidime-avibactam was not detected in any patients during the whole follow-up period. With regard to treatment safety, no adverse events were observed in the study population that were deemed by the treating physicians to be related to ceftazidime-avibactam treatment.

## 3. Discussion

This study is the largest evaluation of a cohort of patients treated with ceftazidime-avibactam for different types of infections due to GNB other than carbapenems-resistant *Enterobacterales*. In line with the pooled clinical cure rate observed in prior trials (85%) [19], about 90% of all assessed patients in our study were deemed an overall treatment success at the end of ceftazidime-avibactam treatment. Notably, this high clinical cure rate was observed despite our study population having a higher prevalence of infections caused by MDR, XDR, or PDR pathogens, underlying comorbidities and use of ceftazidime-avibactam as secondary therapy. Our findings also corroborate previous data reporting renal replacement therapy as a risk factor for clinical failure of ceftazidime-avibactam therapy. Finally, there were no reported adverse events deemed to be related to the drug by the treating physicians.

GNB are common in severe healthcare-associated infections, such as nosocomial pneumonia, bloodstream infection (BSI), or intra-abdominal infection (IAI) [20]. Due to the high proportion of MDR gram-negative pathogens [21], the greatest challenge in managing such infections today is the increased need to use the last-line agents such as carbapenems; thus promoting the selection and spread of more carbapenem-resistant strains. Therefore, the search for alternatives to carbapenems for infections caused by multidrug resistant GNB is a clinical priority.

In the absence of porin deficient mutations or efflux pumps, gram-negative resistance to pivotal antibiotics in our area is mainly mediated by the production of β-lactamases, such as class A (such as ESBL, KPC), class C (AmpC), and some class D enzymes (e.g., OXA 48) [22]. None of these affects ceftazidime-avibactam [23] and this context represents, in our opinion, the situation in which the drug could be used as an alternative to carbapenem for the treatment of MDR gram-negative pathogens [24].

Although real-life experiences of ceftazidime-avibactam for the treatment of CRE is accumulating [9,10,11,12,13,14,15], data regarding its effectiveness and safety for the treatment of other gram-negative infections remain rare [16,17,18] and, to the best of our knowledge, are limited to only three single-center retrospective studies including a total of 20 patients. In the largest of these analyses, Santevecchi et al. [17] reported a clinical success rate of 70% in ten multidrug resistant gram-negative infections treated with ceftazidime-avibactam. In our study, we report an overall clinical success rate of 90%. The high rate of patients receiving continuous or intermittent infusion of ceftazidime-avibactam (40%) as well as combination therapy in 80.5% of patients may have contributed to the clinical success observed in our experience.

Interestingly, we found no significant differences in outcome when analyzed according to primary cultured pathogens, with success rates up to 100% for ESBL-producing Enterobacterales infections. In the case of ESBL strains, old β-lactams/β-lactamase inhibitors, such as piperacillin-tazobactam or amoxicillin-clavulanic acid, were considered for many years as carbapenem-sparing options for infections caused by ESBL-producing Enterobacterales [25,26,27]. However, based on the results from the recent MERINO trial, piperacillin-tazobactam should not be longer considered as an alternative to meropenem for bloodstream infections caused by ESBL strains [28].

Recently, based on in vitro studies, some authors have emphasized the use of ceftazidime-avibactam as a potential carbapenem-sparing treatment for infections due to ESBL−producing *Enterobacterales* [29,30,31]. However, experience in real clinical practice with ceftazidime-avibactam for these infections remains limited. In the present study, including four and two patients with monomicrobial and polymicrobial infections due to ESBL-producing *Enterobacterales*, respectively, we report an excellent clinical success, even higher than the results obtained in the early pivotal trials, that mainly included patients with complicated urinary tract infection (cUTI) or IAI [6,7,8]. Our results, should be confirmed in larger samples to more firmly explore the role of ceftazidime-avibactam for the targeted treatment of patients with infections due to ESBL−producing *Enterobacterales* as a possible carbapenem-sparing agent in selected cases, balancing this possibility with that of reserving it for carbapenem-resistant strains.

Predictors of clinical failure in our study are in accordance with the results of a recent retrospective analysis including patients with CRE infections, which showed that receiving continuous renal replacement therapy was the greatest predictor for clinical failure [12]. From a clinical point of view, we suggest closely monitoring drug serum concentrations in all patients receiving ceftazidime-avibactam for serious gram-negative infections during continuous renal replacement therapy. More data are needed to clarify which is the adequate dosage of ceftazidime-avibactam in patients receiving continuous renal replacement therapy.

One of the most concerning issue related to ceftazidime-avibactam therapy is the rate of recurrence and the appearance of ceftazidime-avibactam resistance during or after treatment [12]. In their study, Santavecchi et al. [17] described the emergence of resistance while on therapy with ceftazidime-avibactam in 2 out of 10 patients (20%) who were treated for 50 and 13 days, respectively. In our study, although with the limitations of the non-standardized collection of follow-up samples, no resistance to ceftazidime-avibactam was detected. In our opinion, further studies are needed to clarify whether combination treatment plays a protective role against the emergence of resistance.

Consistent with previous reports [17,32], we were unable to detect adverse drug events related to ceftazidime-avibactam treatment, even when the drug was administered for a relatively prolonged period of time (median of 13 days (range 2–49 days)). However, due to the retrospective nature of our data we are limited to that information reported in the medical records.

Our study has other limitations that should be addressed. First, this was an observational non-comparative study and thus the typical limitations of this study design apply, including the potential effects of unmeasured data, the lack of a control group, and possible confounding factors. Second, although this is the largest experience reported to date, the limited number of patients reported in this study represents an obvious limitation to the extrapolability of our results. Third, our follow-up period may be considered too short for evaluating recurrence, especially in the case of infections such as primary bacteremia or bone infections. Fourth, due to its retrospective nature, we were not able to collect information about when clinicians obtained susceptibility results to ceftazidime-avibactam.

In conclusion, in this observational study ceftazidime-avibactam showed high clinical cure rates when used for treating serious infections caused by MDR-GNB other than CRE. Further studies remain warranted to more comprehensively evaluate the possible role of ceftazidime-avibactam as a targeted carbapenem-sparing option.

## 4. Materials and Methods

This is a multicenter, retrospective case series of all adult patients who received ceftazidime-avibactam for ≥72 h for documented infections caused by MDR−GNB other than CRE in 13 hospitals located in 9 Italian regions (Friuli Venezia Giulia, Veneto, Lombardia, Piemonte, Emilia Romagna, Liguria, Lazio, Puglia, and Sicilia). The study was conducted from 1 July 2017 to 31 July 2019.

Patients were included in the study if they had a documented infection caused by at least one GNB other than CRE. Patients were excluded if: (i) ceftazidime-avibactam was used for prophylactic purpose; (ii) they had an infection caused by non MDR-GNB according to antibiotic susceptibility testing; or (iii) they had an infection caused by a CRE.

The study was approved by the ethics committee of the coordinating center (Azienda Ospedaliera Universitaria Integrata di Udine).

### 4.1. Definitions and Data Collection

All patients were followed-up for at least 30 days after ceftazidime-avibactam therapy was discontinued. Clinical assessments were determined at the end of the follow-up period. Clinical outcomes were characterized as follows: cure, patients had complete resolution of clinical signs and symptoms related to the infection and/or infection cleared with no positive cultures reported at the end of ceftazidime-avibactam therapy; and failure, lack of clinical response and/or death due to infection and/or recurrent infection.

Documented infection was defined as isolation of MDR−GNB other than CRE in presence of signs and symptoms of infection. MDR, XDR, and PDR were defined according to criteria and Magiorakos et al. [33].

Ceftazidime-avibactam was administered at the standard dosage of 2.5 gm IV q8 h. Dose adjustment was required only for patients with moderate renal dysfunction (creatinine clearance (CLCr) <50 mL/min).

AE related to ceftazidime-avibactam treatment were defined as AE that occurred during the period which elapsed from initiation of ceftazidime-avibactam therapy to 30 days after discontinuation of ceftazidime-avibactam therapy, and that were deemed by the treating physicians to be related to ceftazidime-avibactam treatment (according to medical charts data).

The data collected from medical records included the following: age; sex; ward of stay at the onset of infection; underlying diseases; Charlson comorbidity index [34]; type of infection (according to the Centers for Disease Control and Prevention criteria [35]); presence of sepsis and septic shock (defined according to Sepsis 3 criteria [36]); causative organism and susceptibility test results; other antibiotics administered before, concomitant, and after ceftazidime-avibactam; reasons for ceftazidime-avibactam use; type of ceftazidime-avibactam therapy (first-line vs. second-line therapy; empirical vs. targeted therapy; monotherapy vs. combination therapy), duration of ceftazidime-avibactam therapy; adequateness of source control where applicable (source control was defined as adequate in case of: (i) removal of intravascular catheters in patients with bacteremia; (ii) surgical or radiological drainage of infected fluid collection); adverse events.

Collected data were registered on an electronic case report form using REDCap (Research Electronic Data Capture).

### 4.2. Microbiological Methods

Identification of the organisms was performed at each participating center according to their own local practice. Susceptibility to antibiotics was also reported as interpreted by the local laboratories. Of note: (i) ESBL-producing *Enterobacterales* were phenotypically identified using the following criteria: minimum inhibitory concentration (MIC) ≥2 μg/L for a third-generation cephalosporin or meropenem or MIC increase of ≥3 dilutions when combined with clavulanic acid; (ii) resistance to carbapenems in *Enterobacterales* was defined as imipenem and/or meropenem MIC >4 μg/mL or ertapenem MICs of >0.5 μg/mL [37]; (iii) in all participating centers, MIC values of ceftazidime-avibactam were determined by E-test (bioMérieux, Marcy l’Etoile, France) and interpreted according to the current European Committee on Antimicrobial Susceptibility Testing (EUCAST) clinical breakpoints [37].

### 4.3. Statistical Analysis

Both the primary analysis (description of clinical cure rates in the entire study population and in subgroups according to type of infection and causative agents, using numbers and percentages) and the secondary analysis (description of the characteristics of patients who experienced clinical failure and of adverse events related to ceftazidime-avibactam treatment) were descriptive and the related results were reported in terms of numbers and percentages for categorical data and median and interquartile range (IQR) for continuous data. 

## Figures and Tables

**Table 1:** Baseline demographics and clinical characteristics.

Variables	*n* = 41
Age (years), median (IQR)	62 (41–70)
Sex, male, *n* (%)	28 (68.3)
Ward, *n* (%)	
Medical	17 (41.5)
Surgical	7 (17.1)
Intensive care unit	17 (41.5)
Underlying disease, *n* (%)	
Cardiovascular disease	14 (34.1)
Chronic renal disease	9 (22.0)
Diabetes mellitus	8 (19.5)
Solid organ transplant	8 (19.5)
Neurological disease	7 (17.1)
Solid organ tumors	7 (17.1)
Bronchiectasis	6 (14.6)
Chronic obstructive pulmonary disease	5 (12.2)
Gastrointestinal disease	4 (9.8)
Hematological malignancy	4 (9.8)
Charlson comorbidity index, mean (±SD)	4 (2–6)
Other predisposing conditions ^#^, *n* (%)	
Corticosteroids	12 (29.3)
Chemotherapy	7 (17.1)
Neutropenia (absolute neutrophil count <500 mm^3^)	5 (12.2)
Invasive procedures/devices, *n* (%)	
Central venous catheter	29 (70.7)
Urinary catheter	26 (63.4)
Previous surgery ^#^	15 (36.6)
Mechanical ventilation	14 (34.1)
Percutaneous endoscopic gastrostomy	2 (4.9)
Severity of clinical presentation, *n* (%)	
No sepsis	17 (41.5)
Sepsis	17 (41.5)
Septic shock	7 (17.1)
ICU admission due to the index infection n (%)	10 (24.4)

^#^ Within previous 30 days. Other infections include: 1 Central venous catheter-(CVC) related bacteremia; 1 pyelonephritis, 1 malignant external otitis, and 1 endocarditis. IQR, interquartile range; ICU, intensive care unit.

**Table 2:** Type of primary site of infection and causative agents.

Primary Site of Infection ^&^	Overall	*P. Aeruginosa*	ESBL−Producing *Enterobacterales*	Polymicrobial
Nosocomial pneumonia	20 (48.8)	18	0	2 *
Primary bacteremia	7 (17.1)	5	1	1 ^±^
Intra-abdominal infection	4 (9.8)	2	1	1 ^#^
Bone infection	4 (9.8)	3	1	0
Acute bacterial skin and skin structure infection	2 (4.9)	2	0	0
Other infections ^§^	4 (9.8)	3	1	0
Total	41	33	4	4

^&^ Seven patients (17.1%) had concomitant bacteremia. The portal of entries were lungs (2), abdomen (2), kidney (1), heart (1), and mediastinum (1); * Two mixed ventilator associated pneumonia (VAP) [extended spectrum beta lactamase (ESBL) Enterobacteriaceae + *P. aeruginosa*]; ^±^ One mixed intra-abdominal infection (IAI) due to *P. aeruginosa* and *A. baumannii*; ^#^ One mixed bloodstream infection (BSI) due to *P. aeruginosa* and ESBL producing Enterobacteriaceae; ^§^ Other infections include: 1 CVC related bacteremia; 1 pyelonephritis, 1 malignant external otitis, and 1 endocarditis.

**Table 3:** Susceptibility test results of 45 non-*Enterobacterales (*CRE) multidrug-resistant gram-negative bacteria (MDR-GNB) isolates from 41 patients.

	Non-Susceptible Isolates, *n* (%)		
Antibiotic	Overall (*n* = 45)	*P. Aeruginosa* (*n* = 38)	ESBL-producing *Enterobacterales* (*n* = 7)
**Amikacin**	25 (55.6)	20 (52.6)	5 (71.4)
**Cefepime**	43 (95.6)	36 (94.7)	7 (100)
**Ceftazidime**	40 (88.9)	33 (86.8)	7 (100)
**Ceftolozane-tazobactam**	5 (11.1)	4 (10.5)	1 (14.8)
**Ciprofloxacin**	41 (91.1)	34 (89.4)	7 (100)
**Colistin**	12 (26.6)	12 (31.5)	0
**Gentamycin**	34 (75.6)	29 (76.3)	5 (71.4)
**Imipenem**	35 (77.8)	35 (92.1)	0
**Meropenem**	33 (73.3)	33 (86.8)	0
**Piperacillin-tazobactam**	39 (86.7)	34 (89.4)	5 (71.4)

**Table 4:** Previous treatment characteristics and ceftazidime-avibactam treatment information.

VARIABLE	*n* = 41
**Antibiotics before ceftazidime-avibactam for the current infection**	
**Received antibiotics before ceftazidime-avibactam, *n* (%)**	27 (65.9)
**Number of antibiotics received, median (IQR)**	1 (1–2)
**Days of antibiotic therapy, median (IQR)**	11 (4.5–13)
**Main reason for ceftazidime-avibactam use**	
**Antimicrobial resistance to previous antibiotic**	25 (61.0)
**Previous antibiotic failure**	14 (34.1)
**Previous colonization with carbapenemase-producing microorganisms**	10 (24.4)
**Ceftazidime-avibactam treatment**	
**Targeted therapy**	33 (80.5)
**Empirical therapy**	8 (19.5)
**Combination therapy**	33 (80.5)
**Continuous renal replacement therapy**	5 (12.2)
**Days of treatment, median (range)**	13 (3–49)
**Intermittent infusion**	26 (63.4)
**Continuous infusion**	2 (4.9)
**Extended infusion**	13 (31.7)
**Adequate source control of the infection, *n* (%)**	11/13 (84.6)
**Clinical cure, *n* (%)**	37 (90.2)

**Table 5:** Description of patients who experienced clinical failure.

Age/Sex	Underlying Condition	Type of Infection	Concomitant BSI	Clinical Presentation	Prior Therapy to C/A	Dose of C/A	CRRT	Other Interventions	Reason for Clinical Failure
**73/F**	Wide intestinal resection and hemicolectomy for intestinal obstruction due to metastatic colon cancer; pulmonary embolism; CHF	PDR *P. aeruginosa* Intra−abdominal infection	No	Sepsis	No	1.25 gr/8 h for 8 weeks	Yes.	Inadequate source control of the infection; concomitant colistin therapy	Lack of clinical response
**57/F**	Systemic sclerosis; lung transplant; chronic renal failure	XDR *P. aeruginosa* Nosocomial pneumonia	No	No sepsis	No	2.5 gr/8 h for 10 days	No	No concomitant antibiotics	Lack of clinical response
**41/M**	Burn injury; acute kidney injury	XDR *P. aeruginosa* Primary bacteremia	Yes	Septic shock	No	1.25 gr/8 h for 10 days	Yes	No concomitant antibiotic therapy	Recurrent infection
**76/M**	Diabetes; CHF; urothelial carcinomas	XDR *P. aeruginosa* Nosocomial pneumonia	No	Sepsis requiring ICU admission	Meropenem and amikacin for 5 days	1.25 gr/8 h for 4 days	Yes	Concomitant amikacin	Death

C/A, ceftazidime avibactam; BSI, bloodstream infection; CHF, chronic heart failure; CRRT, continuous renal replacement therapy; PDR, pandrug resistant; XDR, extremely drug resistant.

**Table 6:** Predictors of clinical failure of ceftazidime-avibactam therapy in the study population.

	Univariate Analysis			Multivariate Analysis	
VARIABLE	Successful Clinical Outcome (*n* = 37)	Clinical Failure (*n* = 4)	*p-*Value	OR (95% CI)	*p*-Value
**Age (years), mean ± SD**	56.3 ± 18.4	61.7 ± 16.1	0.59	1.0 (0.88–1.13)	0.96
**Sex, male, *n* (%)**	25 (67.6)	3 (75.0)	1	−	
**Charlson comorbidity index, mean (±SD)**	3.9 ± 3.0	6.2 ± 6.0	0.22	−	
**Underlying disease, *n* (%)**				−	
**Cardiovascular disease**	12 (32.4)	2 (50.0)	0.59	−	
**Chronic renal disease**	8 (21.6)	1 (25.0)	1	−	
**Diabetes mellitus**	7 (18.9)	1 (25.0)	1	−	
**Solid organ transplant**	7 (18.9)	1 (25.0)	1	−	
**Neurological disease**	7 (18.9)	0	1	−	
**Solid organ tumors**	5 (13.5)	2 (50.0)	0.12	6.09 (0.30–123.61)	0.42
**Bronchiectasis**	6 (16.2)	0	1	−	−
**Chronic obstructive pulmonary disease**	5 (13.5)	0	1	−	−
**Gastrointestinal disease**	4 (10.8)	0	1	−	−
**Hematological malignancy**	4 (10.8)	0	1	−	−
**Other predisposing conditions ^#^, *n* (%)**				−	−
**Corticosteroids**	11 (29.7)	1 (25.0)	1	−	−
**Chemotherapy**	7 (18.9)	0	1	−	−
**Neutropenia (absolute neutrophil count <500 mmc3)**	5 (13.5)	0	1	−	−
**Invasive procedures, *n* (%) ^#^**					
**Central venous catheter**	25 (67.6)	4 (100)	0.30	−	−
**Urinary catheter**	23 (62.2)	3 (75.0)	1	−	−
**Previous surgery**	12 (32.4)	3 (75.0)	0.13	−	−
**Mechanical ventilation**	11 (29.7)	3 (75.0)	0.10	3.74 (0.14–95.89)	0.42
**Percutaneous endoscopic gastrostomy**	2 (5.4)	0	1	−	−
**Severity of clinical presentation, n (%)**					
**No sepsis**	16 (43.2)	1 (25.0)	0.14	−	−
**Sepsis**	15 (40.5)	2 (50.0)	1	−	−
**Septic shock**	6 (16.2)	1 (25.0)	0.54	−	−
**Intensive care unit admission due to gram negative infection *n* (%)**	9 (24.3)	1 (25.0)	1	−	−
**Type of infection, *n* (%)**					−
**Nosocomial pneumonia**	18 (48.6)	2 (50.0)	1	−	
**Primary bacteremia**	6 (16.2)	1 (25.0)	0.54	−	−
**Intra-abdominal infection**	3 (8.1)	1 (25.0)	0.34	−	−
**Bone infection**	4 (10.8)	0	1	−	−
**Acute bacterial skin and soft tissue infection**	2 (5.4)	0	1	−	−
**Other infections ^§^**	4 (10.8)	0	1	−	−
**Microorganisms**					
***P. aeruginosa***	342 (81.1)	4 (100)	1	−	−
**Enterobacteriaceae**	7 (18.9)	0		−	−
**C/T treatment**					
**Combination therapy**	31 (83.8)	2 (50.0)	0.16	−	−
**Empirical therapy**	7 (18.9)	1 (25.0)	1	−	−
**Intermittent hemodialysis**	2 (5.4)	2 (25.0)	0.27	−	−
**Continuous renal replacement therapy**	2 (5.4)	3 (75.0)	0.004	29.03 (1.69−498.35)	0.02
**Intermittent Infusion**	23 (62.2)	3 (75.0)	1	−	−
**Continuous infusion**	2 (5.4)	0	1	−	−
**Extended infusion**	12 (32.4)	1 (25.0)	1	−	−
**Adequate source control of the infection, *n* (%)**	9 (81.8)	2 (100)	1	−	−

^#^ Within previous 30 days; ^§^ Other infections include: 1 CVC related bacteremia; 1 pyelonephritis, 1 malignant external otitis, and 1 endocarditis. OR, odds ratio; CI, confidence interval;

## Supplementary Materials

The following are available online at https://www.mdpi.com/2079-6382/9/2/71/s1, Supplementary Materials: Characteristics of patients experiencing clinical failure.
