# Supplementary material for: Clinical Experience with Ceftazidime-Avibactam for the Treatment of Infections due to Multidrug-Resistant Gram-Negative Bacteria Other than Carbapenem-Resistant Enterobacterales
Source: Antibiotics (Basel). 2020 Feb 9;9(2):71. doi: 10.3390/antibiotics9020071 (PMC7168189; doi:10.3390/antibiotics9020071)
Supplement: Supplementary file 1 [file antibiotics-09-00071-s001.pdf]

Supplementary material 1 ***Characteristics of patients experiencing clinical failure***

In two cases, failures occurred because of lack of clinical response. One of the patients had complicated intra-abdominal infection with a late source control (>20 days after infection onset). This patient, who received an 8-weeks course of ceftazidime-avibactam plus colistin, remained asymptomatic only after multiple surgical interventions. The other patient had a nosocomial pneumonia that, after an initial response to ceftazidime-avibactam treatment, experienced respiratory worsening and a new episode of fever. Hence, treatment was switched to ceftolozane-tazobactam plus colistin continued for 14 days with resolution of clinical signs. A third patient had a recurrent bloodstream infection after 5 days from the end of treatment. The strain isolated at recurrence was confirmed to be ceftazidime-avibactam susceptible with no increase in MIC in comparison with the baseline isolate. This patient was retreated with ceftazidime-avibactam in combination with meropenem and was clinically cured. The last case was an old diabetic patient with a pancreatic cancer admitted to the hospital for an episode of ST elevation myocardial infarction. After one month of hospitalization he developed an episode of nosocomial pneumonia due to XDR *P. aeruginosa* requiring ICU admission and orotracheal intubation. Ceftazidime-avibactam plus amikacin were initiated after identification of the causative agent, but on the fourth day of therapy the patient died for refractory septic shock.
